# Anti‐TNF‐Alpha Associated Central Nervous System Demyelination. Drug‐Induced Demyelination or Multiple Sclerosis Trigger? A Case Series

**DOI:** 10.1002/ccr3.73502

**Published:** 2026-09-09

**Authors:** Marzie Abutorabi‐zarchi, Amin Ziyaei, Davoud Ghane

**Affiliations:** ^1^ Shahid Sadoughi Hospital, Shahid Sadoughi Medical School Shahid Sadoughi University of Medical Sciences and Health Services Yazd Iran; ^2^ Student Research Committee Shahid Sadoughi University of Medical Sciences Yazd Iran; ^3^ Shahid Sadoughi Medical School Shahid Sadoughi University of Medical Sciences Yazd Iran

**Keywords:** adalimumab, demyelinating diseases, multiple sclerosis, tumor necrosis factor‐alpha, uveitis

## Abstract

Adalimumab is a monoclonal antibody that targets TNF‐α and is effective in treating several autoimmune disorders, including non‐infectious uveitis; however, it has been associated with demyelinating lesions. This study presents two cases of CNS demyelination in patients who consumed adalimumab. First, an 18‐year‐old man presented with subacute lower‐limb paresis over the past month. He had an 8‐year history of uveitis, which was treated with adalimumab. The patient was initially treated with intravenous methylprednisolone, which resulted in improvement of the symptoms; however, the patient subsequently presented with new symptoms of left lower limb paresis and right homonymous hemianopia. MRI showed periventricular lesions, including two ill‐defined tumefactive demyelinating lesions (TDLs) and a cervical cord lesion. Second, a 35‐year‐old woman with a six‐year history of recurrent inflammatory uveitis, who had been treated with adalimumab every 2 weeks for the last 6 months, developed right‐sided paresthesia and mild weakness. MRI showed classic periventricular “Dawson's fingers” and a short‐segment lesion in the cervical spinal cord. This study demonstrates the diagnostic challenges of demyelination following administration of anti‐TNF‐α agents. Persistence or recurrence of disease activity after discontinuation of adalimumab, along with fulfillment of MS diagnostic criteria, may favor an underlying demyelinating disease rather than a monophasic drug‐induced process. This study emphasizes the importance of careful consideration before prescribing anti‐TNF‐α agents, particularly in patients with risk factors for demyelinating disease.

## Key Clinical Message

Adalimumab is a monoclonal antibody that targets TNF‐α and is utilized to treat several autoimmune disorders; however, it has been associated with the development of demyelinating lesions. This study presents two cases of CNS demyelination after receiving adalimumab and emphasizes the need for careful consideration before prescribing it.

**Abbreviations**
ADEM: Acute Disseminated Encephalomyelitis
CNS: Central Nervous System
IVMP: Intravenous Methylprednisolone
LP: lumbar puncture
MRI: Magnetic Resonance Imaging
MS: Multiple Sclerosis
OCB: Oligoclonal Bands
P/E: plasma exchange
TDL(s): Tumefactive Demyelinating Lesions
TNF‐α: Tumor Necrosis Factor‐alpha

## 1 Introduction

Tumor necrosis factor alpha (TNF‐alpha) is an inflammatory cytokine that plays a role in regulating inflammatory responses in the immune system [1]. In general, TNF‐α functions include activating a series of molecules and inflammatory factors, such as cytokines and chemokines [2]. In the central nervous system (CNS), it plays a part in neurogenesis and myelination of neural cells [3]. Additionally, TNF‐α has a pivotal role in the pathogenesis of Multiple Sclerosis (MS) through its two types of receptors: TNFR1 (TNF receptor 1), which signals neuroinflammatory processes, and TNFR2 (TNF receptor 2), which is engaged in cell survival, neuroprotection, and sustains homeostatic processes [4].

The pro‐inflammatory properties of TNF‐alpha have led to the development of agents that target its activity, commonly referred to as anti‐TNF‐alpha agents. Well‐known examples include adalimumab [5]. Currently, TNF‐alpha inhibitors are used for a wide range of autoimmune diseases [2]. However, their immunomodulatory effects raise concerns about potential adverse neurological events, particularly demyelination of the central nervous system (CNS). In rare cases, these drugs can trigger demyelination in patients with a predisposition or pre‐existing lesions [6, 7].

This study describes two cases of central nervous system demyelination in patients who received adalimumab. Both patients were being treated with adalimumab for a diagnosis of inflammatory uveitis and presented with neurological symptoms. Additionally, this study tries to differentiate between MS triggers and demyelination and also emphasizes the necessity of careful consideration before prescribing the medication.

## 2 Case Presentation

### 2.1 Case 1

#### 2.1.1 Case History/Examination

An 18‐year‐old man presented with subacute lower‐limb paresis over the past month. Neurological examination showed left lower‐limb paresis, paresthesia, and left‐sided hyperreflexia, with bilateral positive Babinski signs. Other neurological examinations were completely normal. There are no systemic symptoms such as arthralgia or skin rash.

The patient's past medical history was significant for recurrent inflammatory uveitis diagnosed 8 years earlier, for which he had been treated with adalimumab, which was initially administered weekly. Still, the dosage was subsequently reduced to once a month over the past year. There was no history of any neurological symptoms or other diseases other than uveitis. At the first visit in our clinic, at the age of seventeen, brain and cervical Magnetic Resonance Imaging (MRI) was done to rule out silent CNS demyelination lesions because of a positive familial history of MS in his mother and treatment with adalimumab, which were completely normal.

The patient initially received intravenous methylprednisolone (IVMP) at a dose of 1 g/day for 5 days, resulting in a significant improvement in his paresis. However, approximately 1 week later, he returned to our clinic with recurrence of left lower limb paresis and new‐onset visual symptoms in the form of acute right homonymous hemianopia.

#### 2.1.2 Differential Diagnosis, Investigations, and Treatment

**FIGURE 1:**
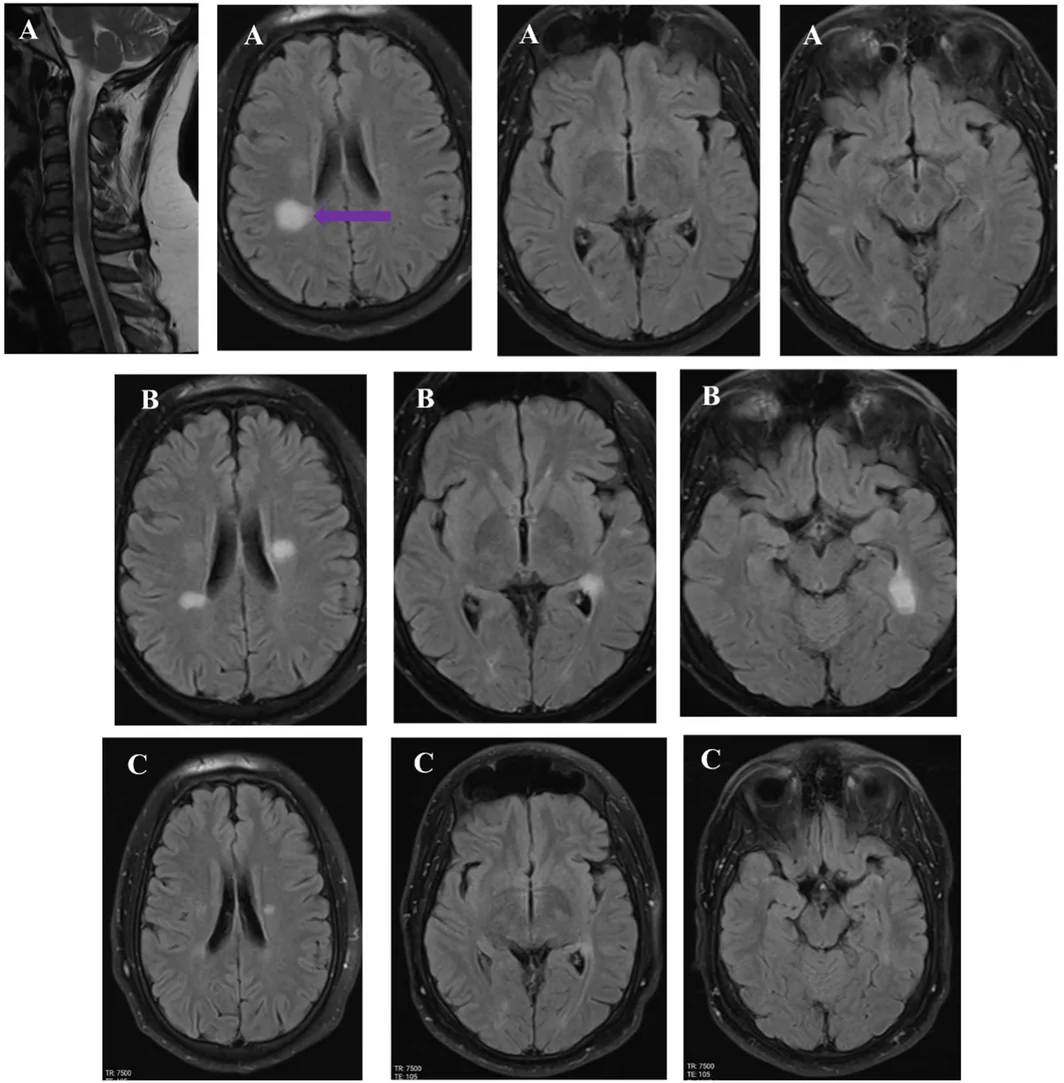
Patient's MRIs: (A) first MRI shows a short‐segment lesion in the cervical cord and periventricular lesions, one of them was TDL marked with an arrow; (B) second MRI after exacerbation of symptoms shows improvement in prior lesions, and the emergence of two new periventricular lesions, one of them was TDL marked with an arrow; and (C) shows a significant reduction in lesion size after 6 months of treatment.

The first MRI showed periventricular and spinal cord demyelinating lesions, as well as ill‐defined tumefactive demyelinating lesions (TDLs). The second MRI, after symptom recurrence, showed improvement in prior lesions and the emergence of two new lesions (Figure 1).

Considering the patient's medical history of anti‐TNF‐α therapy, family history, clinical examination, and MRI findings, the differential diagnoses included inflammatory demyelinating disease such as multiple sclerosis. Other differential diagnoses included a brain tumor, but the lack of vasogenic edema and the existence of a cervical cord lesion were not in favor of a brain tumor. Lab tests, including rheumatological tests including ANA, anti‐dsDNA, PANCA, CANCA, beta‐2 glycoprotein IgG& IgM, Anticardiolipin antibodies IgG& IgM, Anti‐RO/SSA, Anti‐La/SSB, C3, C4, were normal, and Anti‐MOG and anti‐NMO antibodies were also negative. Lumbar puncture for cerebrospinal fluid (CSF) analysis with evaluation of oligoclonal bands (OCBs) was proposed, but the patient and his parents did not permit it in the acute phase, as they were so anxious at the time. We needed their permission to perform the LP (lumbar puncture) based on local legal and ethical rules of medical practice.

IVMP was administered again (1 g/day for 5 days), but due to lack of response, P/E (plasma exchange) for five sessions was started, which led to significant recovery. Rituximab was administered after P/E due to recurrence of symptoms and ongoing demyelinating disease, and adalimumab was discontinued.

#### 2.1.3 Outcome and Follow‐Up

**FIGURE 2:**
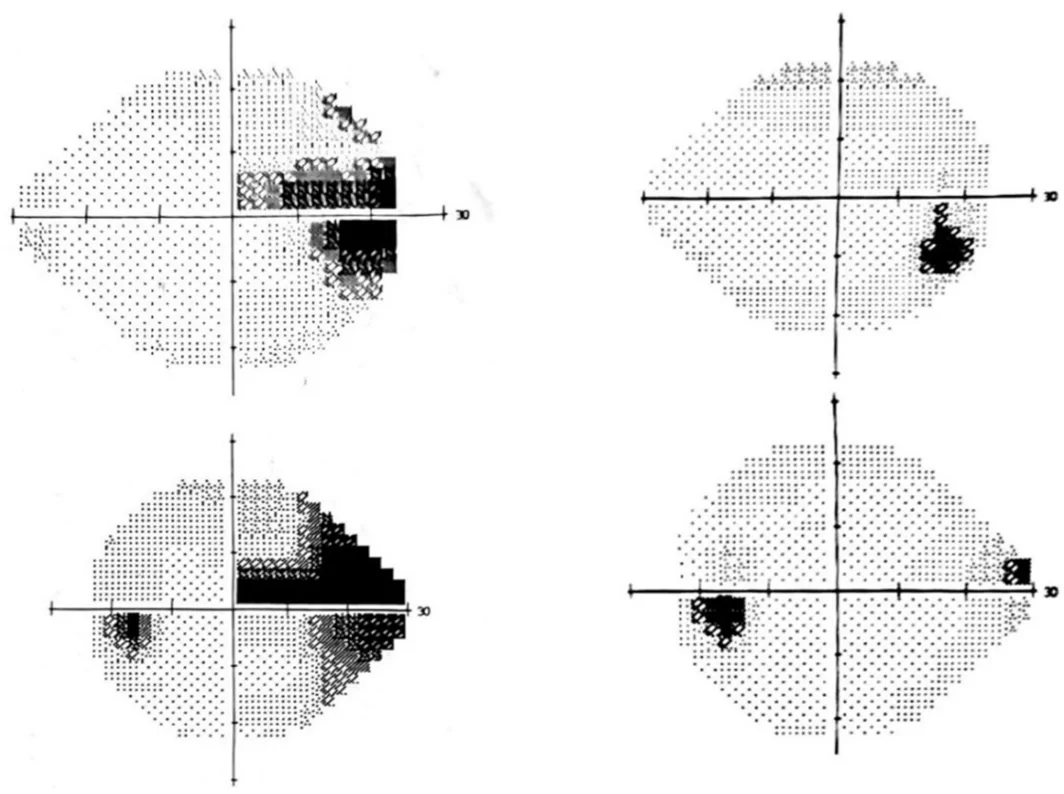
Homonymous hemianopia in a patient (left) and improvement after treatment (right).

Following treatment of the acute phase, the patient's sensorimotor and visual symptoms showed significant improvement after 1 month (Figure 2). Follow‐up imaging also showed a reduction in both the number and size of the demyelinating lesions after 6 months, with stability at 15 months.

After 6 months, when our patient was clinically and paraclinically stable, we explained the importance of checking OCB for the diagnosis of MS to the patient and his parents again, and this led to their consent to undergo the LP to check for OCB, which was positive.

Although TDL and homonymous hemianopia are atypical for MS, overall, with ruling out other diseases and positive OCB, the diagnosis of MS was established based on the 2024 McDonald criteria. Dissemination in space was fulfilled by periventricular and cervical cord lesions (two of the five typical locations), and dissemination in time was completed with positive OCB [8].

Rituximab 1 g every 6 months was continued for maintenance therapy of multiple sclerosis as well as control of uveitis. Rituximab is used routinely as second‐line disease‐modifying therapy (DMT) in our country based on the MENACTRIMS guideline [9]. During the 15‐month follow‐up period, there is no evidence of relapse or MRI activity. Patients' disease and treatment timeline is presented in Table 1.

**TABLE 1:** Disease and treatment timeline in case 1.

Diagnosis of uveitis and start of adalimumab	10/3/2016
Normal brain and cervical MRI	14/3/2024
Onset of neurological symptom	14/3/2025
First MRI	14/4/2025
First IVMP therapy	15/4/2025–20/4/2025
Recurrence of weakness and new visual symptoms	27/4/2025
Second MRI	29/4/2025
Second IVMP therapy	30/4/2025–4/5/2025
Plasma exchange	5/5/2025–15/5/2025
Administration of the first dose of rituximab	16/5/2025
Checking of OCB and confirmed MS	11/2025
Administration of the second dose of rituximab	17/11/2025
First follow‐up MRI	17/11/2025
Administration of second dose of rituximab	18/5/2026
Second follow‐up MRI	15/6/2026

### 2.2 Case 2

#### 2.2.1 Case History/Examination

A 35‐year‐old woman presented with acute right hemiparesthesia and hemiparesis. The patient's past medical history included a history of recurrent inflammatory uveitis for the past 6 years, which was treated with several immunosuppressive drugs. During the 6 months preceding the onset of neurological symptoms, the patient had been treated with low‐dose prednisolone in combination with adalimumab. Also, before this presentation, the patient had no history of other neurological symptoms or diseases other than uveitis. On neurological examination, the patient demonstrated mild weakness with reduced muscle strength (4/5) accompanied by sensory disturbances on the right side of the body with bilateral positive hoffmann`s sing and right hyperreflexia and positive babinski. Other neurological assessments were within normal limits.

#### 2.2.2 Differential Diagnosis, Investigations, and Treatment

**FIGURE 3:**
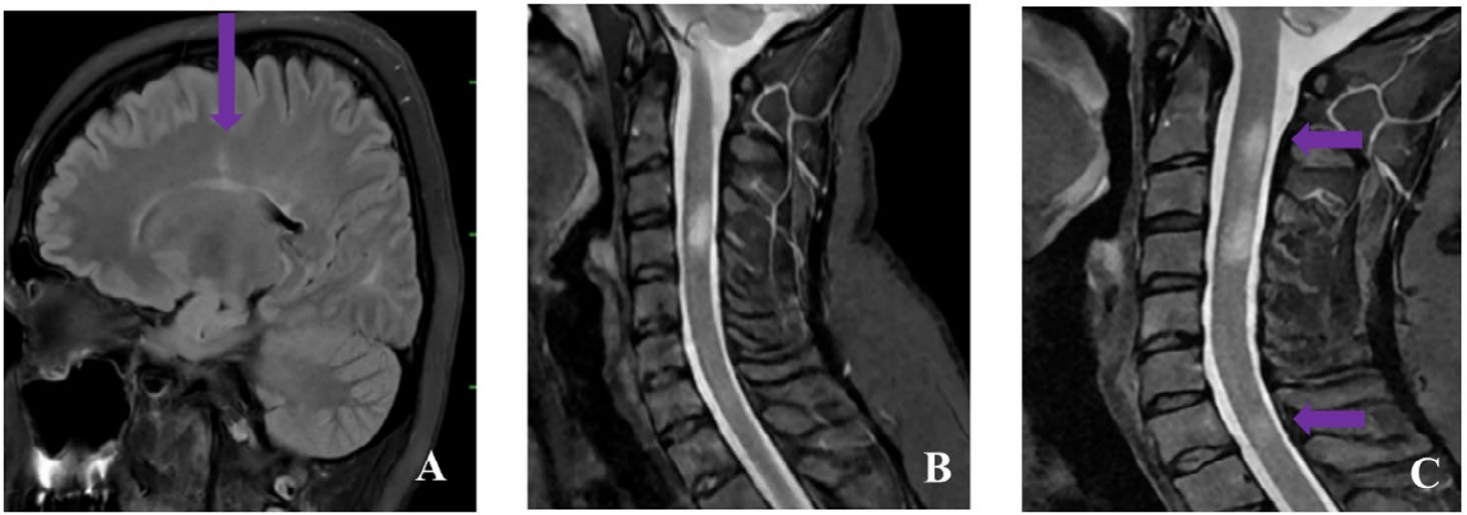
Brain MRI with typical MS lesions (A) and multiple periventricular lesions (Dawson's fingers sign) marked with an arrow (B). Short segment lesion in cervical MRI (C) and two new short segment lesions in the cervical cord after 5 months marked with arrows.

Brain and spinal MRI were requested for further evaluation. Brain MRI demonstrated periventricular lesions with a “Dawson's fingers” morphology. In addition, spinal MRI revealed demyelinating lesions in the cervical spinal cord (Figure 3). Overall, the radiological findings were highly suggestive of multiple sclerosis. Based on the clinical presentation and MRI findings, the differential diagnoses included multiple sclerosis, other demyelination, and, less likely, other inflammatory diseases.

Rheumatological lab tests, including ANA, anti‐dsDNA, PANCA, CANCA, beta‐2 glycoprotein IgG & IgM, Anticardiolipin antibodies IgG & IgM, Anti‐RO/SSA, Anti‐La/SSB, C3, and C4, were normal. Given the typical MS lesions on brain MRI and the presence of a short‐segment peripheral lesion on spinal cord MRI, inflammatory disorders associated with longitudinally extensive transverse myelitis (LETM), such as neuromyelitis optica spectrum disorder (NMOSD) and myelin oligodendrocyte glycoprotein antibody‐associated disease (MOGAD), were not considered, and the corresponding antibodies, including anti‐AQP4 and anti‐MOG antibodies, were not tested.

After the diagnosis was established, adalimumab was discontinued. The patient was subsequently treated with IVMP (1 g/day for 5 days), which resulted in significant improvement of neurological symptoms. Following rheumatology consultation, low‐dose prednisolone and azathioprine were initiated for simultaneous control of uveitis and demyelinating disease.

#### 2.2.3 Outcome and Follow‐Up

Five months later, the patient re‐presented with new‐onset sensory disturbances. Repeat imaging was performed, and MRI of the cervical spinal cord revealed tow new lesions. The patient was again treated with IVMP. In addition, azathioprine was discontinued and replaced with Rituximab. Considering typical MS lesions and relapses with new cervical cord lesions, the diagnosis of MS was established based on the 2024 McDonald criteria. Dissemination in space was fulfilled by periventricular and cervical cord lesions (two of the five typical locations), and dissemination in time was completed with relapse and a new lesion [8]. We proposed to start rituximab for treatment of MS and uveitis concurrently and discontinued azathioprine. The patient did not return to the clinic afterward, and further follow‐up is not available. The patient's disease and treatment timeline is presented in Table 2.

**TABLE 2:** Disease and treatment timeline in case 2.

Diagnosis of uveitis	18/5/2019
Start of adalimumab	18/11/2024
Onset of neurological symptom	5/5/2025
First MRI	15/5/2025
First IVMP therapy	18/5/2025–21/5/2025
Start of azathioprine	22/5/2025
New‐onset sensory symptom	25/10/2025
Second MRI and confirmed MS diagnosis	29/10/2025

## 3 Discussion

Anti‐TNF‐alpha drugs are used to treat inflammatory diseases. In diseases such as non‐infectious uveitis, like the cases presented in this study, paradoxically, these drugs can trigger demyelination.

In the first case, although homonymous hemianopia and TDL are atypical for MS [6], positive OCB helped diagnose MS. OCB can be transiently positive in non‐MS demyelinating lesions such as acute disseminated encephalomyelitis (ADEM) [10] in the acute phase. Positive OCB 6 months after the acute phase suggests MS (a non‐monophasic demyelinating process) rather than monophasic demyelinating processes such as ADEM or drug‐induced demyelination. However, given the long‐term use of adalimumab, the dose reduction over the past year, and the persistence of disease activity after discontinuing the medication, this process appears independent of the drug. Although a positive family history of MS, it should be considered that initiating anti‐TNF‐alpha agents is not recommended in individuals with a positive family history of MS. These medications are better replaced with another drug.

In the second case, diagnosis of MS was established based on the patient's MRI that showed lesions typical of MS, and relapse after 5 months from discontinuation of the anti‐TNF‐α drug, but due to recent use of adalimumab, the drug may play a role in clinically triggering the disease, rather than causing it.

Uveitis can also occur in patients with MS and be a precursor to it [11]. MS‐related uveitis or demyelinating plaque‐associated uveitis is often characterized by bilateral intermediate uveitis with peripheral vascular leakage or panuveitis [12]. Patients with (any type of) uveitis are approximately three times more likely to develop MS during 15 years of follow‐up compared to a similar control group [13]. Given the above, it is recommended that, in cases of inflammatory uveitis without a specific rheumatologic cause, CNS demyelination be investigated with a CNS MRI. Based on recurrent neurological symptoms after discontinuation of the anti‐TNF‐α drug, the treatment of the patient was followed with Rituximab, which is effective for MS or demyelinating lesions and non‐infectious uveitis [14].

In vitro, studies have shown that TNF‐α induces mitochondrial dysfunction in oligodendrocyte progenitor cells, ultimately leading to impaired oligodendrocyte precursor differentiation and reduced axonal myelination [15]. However, trials of anti‐TNF agents for MS patients have shown that they are not only ineffective but also have adverse effects, including activation of the immune system and exacerbation of the disease [16]. Cases of MS being triggered following the use of TNF‐alpha inhibitors have been reported in the past. Also, in rare cases, the new onset of MS has occurred following the use of this drug.

In conclusion, it is also advisable to avoid the administration of anti‐TNF‐alpha agents to patients with MS and those with a positive family history if it is possible. For other patients, an MRI scan of the central nervous system to exclude subclinical demyelinating lesions can support more informed decisions about initiating TNF‐α inhibitor therapy and potentially prevent adverse effects. Anti‐TNFα should also be discontinued after CNS demyelination occurs. Ultimately, it is recommended that, in cases of inflammatory uveitis without a specific inflammatory cause, CNS demyelination be investigated with a CNS MRI.

## Author Contributions

**Marzie Abutorabi‐zarchi:** conceptualization, supervision, writing – original draft, writing – review and editing, investigation, data curation. **Amin Ziyaei:** writing – original draft, writing – review and editing. **Davoud Ghane:** data curation, investigation.

## Funding

The authors have nothing to report.

## Ethics Statement

This study was approved by the Ethics Committee of Shahid Sadoughi University of Medical Sciences, Yazd, Iran (Approval code: IR.SSU.REC.1404.053).

## Consent

Written informed consent from patients was obtained for publication of their clinical information and medical images. The patients' identifying information has been removed to ensure their privacy.

## Conflicts of Interest

The authors declare no conflicts of interest.

## Data Availability

The data of this study are available from the corresponding author upon reasonable request.

## References

[1] J. R. Bradley , “TNF‐Mediated Inflammatory Disease,” Journal of Pathology 214, no. 2 (2008): 149–160, 10.1002/path.2287. 18161752

[2] D. I. Jang , A. H. Lee , H. Y. Shin , et al., “The Role of Tumor Necrosis Factor Alpha (TNF‐α) in Autoimmune Disease and Current TNF‐α Inhibitors in Therapeutics,” International Journal of Molecular Sciences 22, no. 5 (2021): 2719, 10.3390/ijms22052719. 33800290 PMC7962638

[3] N. Gonzalez Caldito , “Role of Tumor Necrosis Factor‐Alpha in the Central Nervous System: A Focus on Autoimmune Disorders,” Frontiers in Immunology 14 (2023): 1213448, 10.3389/fimmu.2023.1213448. 37483590 PMC10360935

[4] V. Mazziotti , F. Crescenzo , E. Turano , et al., “The Contribution of Tumor Necrosis Factor to Multiple Sclerosis: A Possible Role in Progression Independent of Relapse?,” Journal of Neuroinflammation 1, no. 1 (2024): 209, 10.1186/s12974-024-03193-6. PMC11340196 39169320

[5] T. Horiuchi , H. Mitoma , S. Harashima , H. Tsukamoto , and T. Shimoda , “Transmembrane TNF‐Alpha: Structure, Function and Interaction With Anti‐TNF Agents,” Rheumatology (Oxford, England) 49, no. 7 (2010): 1215–1228, 10.1093/rheumatology/keq031. 20194223 PMC2886310

[6] R. Alnasser Alsukhni , Z. Jriekh , and Y. Aboras , “Adalimumab Induced or Provoked MS in Patient With Autoimmune Uveitis: A Case Report and Review of the Literature,” Case Reports in Medicine 2016 (2016): 1423131, 10.1155/2016/1423131. 27840642 PMC5093248

[7] Z. Al Obaidi and N. Anadani , “Diverse CNS Manifestations Related to TNF Inhibitors: A Case Series Review,” Neurology 103, no. 7_Supplement_1 (2024): S78, 10.1212/01.wnl.0001051512.22392.a8.39378381

[8] X. Montalban , C. Lebrun‐Frénay , J. Oh , et al., “Diagnosis of Multiple Sclerosis: 2024 Revisions of the McDonald Criteria,” Lancet Neurology 24, no. 10 (2025): 850–865, 10.1016/S1474-4422(25)00270-4. 40975101

[9] B. Yamout , M. Al‐Jumah , M. A. Sahraian , et al., “Consensus Recommendations for Diagnosis and Treatment of Multiple Sclerosis: 2023 Revision of the MENACTRIMS Guidelines,” Multiple Sclerosis and Related Disorders 83 (2024): 105435, 10.1016/j.msard.2024.105435. 38245998

[10] A. Javed and O. Khan , “Acute Disseminated Encephalomyelitis,” Handbook of Clinical Neurology 123 (2014): 705–717, 10.1016/b978-0-444-53488-0.00035-3. 25015513 PMC7151857

[11] E. Raskin , A. Achiron , O. Zloto , R. Neuman , and V. Vishnevskia‐Dai , “Uveitis Prior to Clinical Presentation of Multiple Sclerosis (MS) is Associated With Better MS Prognosis,” PLoS One 17, no. 6 (2022): e0264918, 10.1371/journal.pone.0264918. 35767533 PMC9242474

[12] A. Hedayatfar , P. Anvari , C. P. Herbort , and S.‐P. Chee , “Demyelinating Plaque‐Associated Uveitis,” Graefe's Archive for Clinical and Experimental Ophthalmology 262, no. 2 (2024): 575–582, 10.1007/s00417-023-06270-3. 37855958

[13] C. Heppell , A. Subramanian , N. J. Adderley , et al., “Comprehensive Update on Multiple Sclerosis‐Associated Uveitis and New Epidemiological Insights From the United Kingdom,” Ocular Immunology and Inflammation 33, no. 4 (2025): 535–547, 10.1080/09273948.2025.2491567. 40238829

[14] H. Cao and X. Ma , “Rituximab in the Treatment of Non‐Infectious Uveitis: A Review,” Journal of Inflammation Research 17 (2024): 6765–6780, 10.2147/jir.S477708. 39364118 PMC11448468

[15] M. Bonora , E. De Marchi , S. Patergnani , et al., “Tumor Necrosis Factor‐α Impairs Oligodendroglial Differentiation Through a Mitochondria‐Dependent Process,” Cell Death and Differentiation 21, no. 8 (2014): 1198–1208, 10.1038/cdd.2014.35. 24658399 PMC4085526

[16] M. Zahid , A. Busmail , S. S. Penumetcha , et al., “Tumor Necrosis Factor Alpha Blockade and Multiple Sclerosis: Exploring New Avenues,” Cureus 13, no. 10 (2021): e18847, 10.7759/cureus.18847. 34804701 PMC8597935

